# Identification of proteins influencing CRISPR-associated transposases for enhanced genome editing

**DOI:** 10.1126/sciadv.aea1429

**Published:** 2026-01-01

**Authors:** Leo C. T. Song, Amanda T. P. Alker, Agnès Oromí-Bosch, Sophia E. Swartz, Jonathan N. V. Martinson, Jigyasa Arora, Abby M. Wang, Rachel Rovinsky, Sara J. Smith, Emily C. Pierce, Adam M. Deutschbauer, Jennifer A. Doudna, Brady F. Cress, Benjamin E. Rubin

**Affiliations:** ^1^ Innovative Genomics Institute, University of California, Berkeley, Berkeley, CA 94720, USA.; ^2^ Department of Comparative Biochemistry, University of California, Berkeley, Berkeley, CA 94720, USA.; ^3^ Department of Biomedical and Pharmaceutical Sciences, University of Rhode Island, Kingston, RI 02881, USA.; ^4^ Department of Molecular and Cell Biology, University of California, Berkeley, Berkeley, CA 94720, USA.; ^5^ Department of Bacteriology, University of Wisconsin-Madison, Madison, WI 53706, USA.; ^6^ Department of Medicine, Division of Rheumatology, University of California, San Francisco, San Francisco, CA 94143, USA.; ^7^ CoLabs, University of California, San Francisco, San Francisco, CA 94143, USA.; ^8^ Division of Biological Sciences, University of California, San Diego, La Jolla, CA 92093, USA.; ^9^ Environmental Genomics and Systems Biology Division, Lawrence Berkeley National Laboratory, Berkeley, CA 94720, USA.; ^10^ Howard Hughes Medical Institute, University of California, Berkeley, Berkeley, CA 94720, USA.; ^11^ California Institute for Quantitative Biosciences, University of California, Berkeley, Berkeley, CA 94720, USA.; ^12^ Gladstone Institutes, San Francisco, CA 94158, USA.; ^13^ Molecular Biophysics and Integrated Bioimaging Division, Lawrence Berkeley National Laboratory, Berkeley, CA 94720, USA.; ^14^ Department of Chemistry, University of California, Berkeley, Berkeley, CA 94720, USA.

## Abstract

CRISPR-associated transposases (CASTs) hold tremendous potential for microbial genome editing because of their ability to integrate large DNA cargos in a programmable, site-specific manner. However, their widespread application has been hindered by poorly understood host factor requirements for transposition. To address this gap, we conducted the first genome-wide screen for host factors affecting *Vibrio cholerae* CAST (*Vch*CAST) activity using an *Escherichia coli* RB-TnSeq library and identified 15 genes affecting *Vch*CAST transposition. Of these, seven factors were validated to improve *Vch*CAST activity, and two were inhibitory. Guided by the identification of homologous recombination effectors, RecD and RecA, we tested the λ-Red recombineering system in our *Vch*CAST editing vectors and increased editing efficiency by 55.2-fold in *E. coli*, 5.6-fold in *Pseudomonas putida*, and 10.8-fold in *Klebsiella michiganensis* while maintaining high target specificity and similar insertion arrangements. This study improves the understanding of factors affecting *Vch*CAST activity and enhances its efficiency as a bacterial genome editor.

## Teaser

Whole-genome screening reveals strategies to boost CRISPR-associated transposase genome editing efficiency.

## INTRODUCTION

CRISPR-associated transposases (CASTs) represent a powerful recent addition to the genome editing toolbox (*1*). Traditional CRISPR-Cas systems create double-stranded breaks to facilitate editing, which are often lethal in bacteria (*2*). In contrast, CASTs combine RNA-guided targeting with transposon 7 (Tn*7*)–like transposition to enable large programmable insertions without this limitation (*3*). Unlike homologous recombination, a standard alternative for microbial editing that requires lengthy homology arms, CASTs are programmed with short 24– to 32–base pair (bp) guides (depending on the system) that can be multiplexed for simultaneous edits (*4*, *5*). Among the currently found and tested CASTs—including those with a single Cas component (type V) and multiple Cas components (type I)—the type I systems are significantly more accurate in *Escherichia coli* (*6*–*8*). Within this group, the type I-F *Vibrio cholerae* CAST (*Vch*CAST) Tn*6677* (fig. S1A) has emerged as a valuable platform for bacterial editing (*9*), manipulating microbial communities (*8*), and even modifying human genomes (*10*).

Despite its established value as a genome editing tool, *Vch*CAST currently faces limitations in achieving efficient editing across a broad range of organisms. For example, in the industrially important species *Corynebacterium glutamicum*, *Vch*CAST achieved an editing efficiency between 0 and 0.027% (*11*). In complex gut microbiome samples, *Vch*CAST has shown editing efficiencies as low as 0.001% when delivered by conjugation (*8*), significantly limiting its utility for in situ microbiome engineering. These low efficiencies restrict *Vch*CAST’s potential for environmental, industrial, and therapeutic genome editing applications, highlighting the need for optimization.

Broader and more efficient use of *Vch*CAST would be facilitated by an understanding of the host factors that enhance and restrict its function. For instance, the addition of caseinolytic mitochondrial matrix peptidase chaperone subunit X (ClpX) was found to facilitate *Vch*CAST functionality in human embryonic kidney 293T cells (*10*); however, ClpX has not been found necessary for its function in bacteria. Within bacteria, integration host factor (IHF) and, to a lesser degree, factor for inversion stimulation (FIS) are the only proteins known to affect *Vch*CAST function (*12*). In addition, CAST editing efficiency rates differ between endogenous hosts and *E. coli* recipients, suggesting more, yet still unidentified, molecular requirements of CAST integration (*13*). A notable opportunity remains for systematic identification of activators and inhibitors for *Vch*CAST transposition.

In this study, we conducted a genome-wide screen to identify host factors that affect *Vch*CAST activity, resulting in the validation of seven activators and two inhibitors. Informed by the identification of homologous recombination-involved effectors, RecD and RecA, we explored whether incorporating the efficient phage-derived λ-Red recombination system could enhance *Vch*CAST editing performance (fig. S1B). By integrating the λ-Red genes into our *Vch*CAST vectors, we achieved a 55.2-fold increase in editing efficiency in *E. coli*. This strategy also facilitated more efficient integration in other industrially, environmentally, and medically relevant bacteria, such as *Pseudomonas putida* by 5.6-fold and *Klebsiella michiganensis* by 10.8-fold. Our research advances the understanding of CAST systems, revealing key regulatory factors and developing strategies to enhance editing efficiency.

## RESULTS

### Whole-genome mutant screen and validation to identify *Vch*CAST regulators

**Fig. 1.**
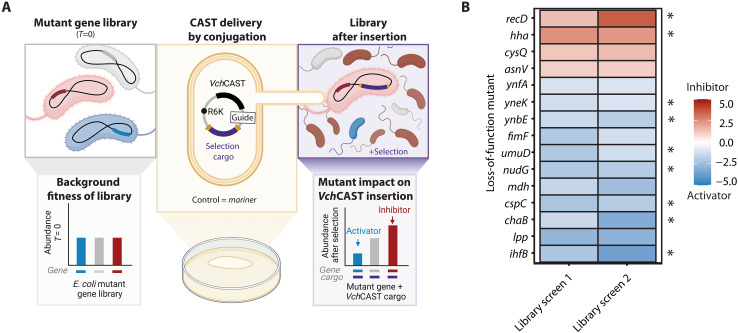
Genome-wide screen identifies putative inhibitors and activators of *Vch*CAST integration. (**A**) Schematic of the RB-TnSeq screen to identify *E. coli* genes affecting *Vch*CAST integration efficiency. Created in BioRender. A. T. P. Alker (2025); https://biorender.com/j90j989. (**B**) Fitness scores of *E. coli* genes from two independent RB-TnSeq screens with fitness scores >1 or <−1 in both screens. Library screens 1 and 2 refer to repeated screens with gentamicin and chloramphenicol selection cargos, respectively. Asterisks denote candidate mutants that were experimentally validated.

We performed a genome-wide survey of genes involved with *Vch*CAST function by screening a library of loss-of-function mutants for the ability of *Vch*CAST to integrate into a safe site. The screen was conducted with a preexisting *E. coli* random bar code transposon-site sequencing (RB-TnSeq) transposon mutant library (*14*). *Vch*CAST was introduced to the library via conjugation, and the inserted cargo was selected in liquid media overnight, ensuring that all members of the library contained both the *Vch*CAST and background loss-of-function mutation (Fig. 1A). The efficiency of *Vch*CAST insertion into each mutant was determined by sequencing the unique barcode of each insertion-containing mutant after outgrowth and comparing its abundance to that of a *mariner* transposase control (see Materials and Methods). *Mariner* was chosen as an unrelated transposon system to control for screening hits that affect delivery efficiency and variables beyond *Vch*CAST integration itself.

We identified 11 genes as putative activators and 4 as putative inhibitors on the basis of consistent absolute gene fitness >1 across the two parallel screens (Fig. 1B). We report the raw fitness scores (table S1) and compare the distribution of log_2_ fold change for *Vch*CAST versus the *mariner* control across both screens (figs. S2 and S3) to inform gene selection for further validation. Notably, the β subunit of IHF (*ihfB*) emerged as the top activator from the screen (Fig. 1B). The other subunit, *ihfA*, did not have a high enough starting library abundance in screen 1 to be considered for our evaluation pipeline (table S2) but, in screen 2, had a comparably low fitness score to *ihfB*. This finding supports previous research identifying IHF as a strong activator of *Vch*CAST insertion (*12*), lending credibility to the screen’s results.

**Fig. 2.**
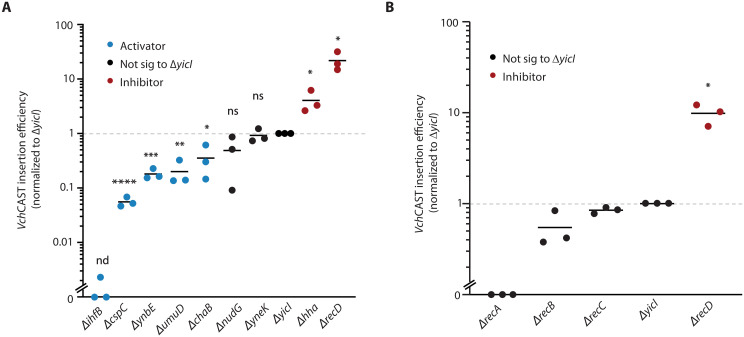
Validation of putative *Vch*CAST activators and inhibitors in single deletion mutants. (**A**) Relative *Vch*CAST editing efficiency in *E. coli* Keio collection mutants of candidate regulators identified from the RB-TnSeq screen. Editing efficiencies were normalized to the Δ*yicI* neutral fitness control strain. Mutants of screen-identified activators with significantly reduced editing efficiency relative to Δ*yicI* are shown in blue, and inhibitors are shown in red [with ihfB previously described (*12*)]; mutants of factors with nonsignificant (ns) difference in editing efficiency compared to Δ*yicI* are denoted by black. nd, not determined. (**B**) Relative *Vch*CAST editing efficiency in *E. coli* Keio collection mutants of RecBCD complex components and downstream homologous recombination effector RecA normalized to the Δ*yicI* negative control strain. Data values at zero represent samples with no viable colonies above the detection limit. Asterisks denote the degree of significance (one sample *t* test) for treatments compared to the ∆*yicI* control (*P ≤ 0.05, **P ≤ 0.006, ***P ≤ 0.0008, and ****P ≤ 0.0001; *n* = 3 biological replicates).

We validated hits with the largest absolute fitness values from the pooled RB-TnSeq screens by testing *Vch*CAST editing in clonal gene deletion mutants. To prioritize candidates most likely to have a direct interaction with *Vch*CAST, we filtered for those having DNA-interacting, RNA-interacting, and protein-interacting functions as well as hypothetical proteins, as per the Gene Ontology database (table S3). We then performed conjugation assays to quantify the relative editing efficiency of *Vch*CAST in Keio deletion mutants of each of the nine filtered hits (Fig. 2A). Editing efficiencies were compared to Δ*yicI*, a deletion mutant with no documented adverse fitness effects (*15*). Five of the mutants of putative activators (*ihfB*, *cspC*, *ynbE*, *umuD*, and *chaB*) exhibited significantly reduced *Vch*CAST editing efficiency (64.6 ± 19.4 to 99.9 ± 0.1%; normalized to the ∆*yicI* neutral fitness control at 100%) (Fig. 2A). Two of the putative activators, *nudG* and *yneK*, were not statistically significant compared to the ∆*yicI* control. Knockouts of putative inhibitors (*hha* and *recD*) exhibited a significant increase in editing efficiency (4.0 ± 1.6–fold and 21.8 ± 7.2–fold) in comparison to the control (Fig. 2A). The results from the whole-genome screen and the individual mutant validation were largely consistent, with *recD* as the strongest inhibitor and *ihfB* as the strongest activator in both experiments. Together, the validation results support the potential involvement of these host factors in *Vch*CAST’s genomic integration.

We were particularly compelled by the strong inhibitor classification of *recD* in *Vch*CAST insertion revealed by the screen and validation and sought to explore the *rec* genes further. RecD inhibits RecBCD-mediated homologous recombination (*16*), suggesting a beneficial role of factors involved in DNA repair during *Vch*CAST integration. To test this hypothesized role, we delivered *Vch*CAST via conjugation into Keio knockout mutants of select, key *rec* genes (*recABCD*) and quantified editing efficiency normalized to Δ*yicI* (Fig. 2B). Consistent with the screen results, the putative inhibitor Δ*recD* showed a 9.8 ± 2.6–fold increase in editing efficiency compared to the neutral fitness mutant control. Knockouts of *recB* and *recC* slightly decreased insertion efficiency relative to the control (45.3 ± 25.5 and 15.0 ± 6.6%, respectively). Δ*recA*, which was not present in the starting library (*14*), had no viable transconjugant colonies above the limit of detection [1 × 10^5^ CFU (colony-forming units)/ml]. The results of this experiment identify *recA* as an activator and, in combination with the identification of *recD* as an inhibitor, support the role of RecBCD-mediated homologous recombination in promoting *Vch*CAST integration.

### Further evidence suggesting homologous recombination’s role in *Vch*CAST integration

Informed by RecA and RecD’s Keio validation results, we sought to further support homologous recombination’s involvement in *Vch*CAST integration by assessing *Vch*CAST’s editing efficiency in cells exposed to ciprofloxacin (CIP). This fluoroquinolone antibiotic has been shown to stimulate homologous recombination in *E. coli* at sublethal concentrations by inducing DNA damage and, consequently, the SOS response (*17*–*19*). We hypothesized that stimulating homologous recombination during *Vch*CAST transposition would increase the efficiency of transposition. BW25113 *E. coli* recipients incubated with the minimum inhibitory concentration (MIC) of CIP before conjugation showed a 58.0 ± 16.9–fold increase in *Vch*CAST editing efficiency compared to the no-CIP control (fig. S4A). Similarly, BW25113 recipients incubated with one-half MIC CIP saw a 25.6 ± 11.9–fold increase in *Vch*CAST editing efficiency compared to the control (fig. S4A). Whole-genome sequencing verified that the edits in both MIC CIP– and one-half MIC CIP–treated *E. coli* were 100% on-target and followed similar insertion distribution patterns as untreated *E. coli* (fig. S4, B and C), indicating that CIP exposure does not affect *Vch*CAST on-target specificity. These results provide further evidence in support of the positive correlation between homologous recombination frequency and *Vch*CAST integration.

### Leveraging λ-Red to improve *Vch*CAST editing efficiency in *E. coli*

**Fig. 3.**
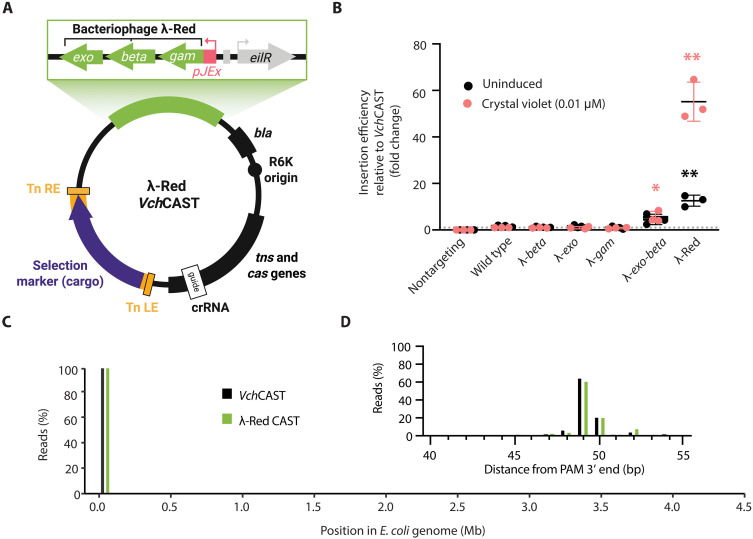
The λ-Red recombineering system improves *Vch*CAST editing efficiency in *E. coli*. (**A**) Schematic of the λ-Red *Vch*CAST vector design. Created in BioRender. A. T. P. Alker (2025); https://biorender.com/o37n467. (**B**) Relative editing efficiency of VchCAST vectors testing the different λ-Red genes (*beta*, *exo*, and *gam*). The editing efficiency of λ-Red *Vch*CAST as well as the nontargeting biological replicates was normalized to the paired *Vch*CAST biological replicates. Asterisks denote statistical significance determined by a one sample *t* test compared to hypothetical mean = 1 of *Vch*CAST grown on CV (one-tailed *P* values: *P ≤ 0.05 and **P ≤ 0.01; *n* = 3 biological replicates). (**C**) On-target insertion frequency of *E. coli* transconjugants via whole-genome sequencing. (**D**) Whole-genome sequencing read distribution (%) of insertions downstream (base pairs) of the PAM target site.

Considering the role of the RecBCD complex and potentially homologous recombination more broadly in *Vch*CAST integration, we hypothesized that introducing higher-efficiency recombination machinery may improve editing outcomes. To this end, the bacteriophage λ-Red genes (*exo, beta,* and *gam*) under the inducible Jungle Express promoter (pJEx) were cloned into a *Vch*CAST plasmid (R6K, P_Pmtl_-*catP* cargo), which does not replicate in *pir−* recipient strains (Fig. 3A). We optimized the induction level of λ-Red by crystal violet (CV) (fig. S5) for editing in *E. coli*. The induced λ-Red *Vch*CAST treatment significantly increased editing efficiency by 55.2 ± 6.9–fold in BW25113 *E. coli* compared to the *Vch*CAST control (Fig. 3B).

To determine which components of λ-Red are responsible for the increase in *Vch*CAST efficiency, we also tested constructs containing inducible *exo* only, *beta* only, *gam* only, and *exo-beta* for their effect on *Vch*CAST insertion. Exo and Beta promote homologous recombination through their exonuclease and single-stranded annealing activity, respectively, while Gam inhibits RecBCD by binding to and blocking the DNA-interacting domain of RecB (*20*–*22*). We found that while the induced *exo*-*beta* construct does improve editing efficiency relative to the *Vch*CAST control by 5.6 ± 2.0–fold in *E. coli*, it does not improve editing to the extent of all three λ-Red genes (55.2 ± 6.9–fold) (Fig. 3B). The presence of *exo*, *beta*, or *gam* alone on the *Vch*CAST vector did not affect editing efficiency (Fig. 3B). These results suggest that the complete system is required to realize the full impact of λ-Red on editing efficiency in *Vch*CAST.

Whole-genome sequencing analysis confirmed that 100% of λ-Red *Vch*CAST insertions were on target in *E. coli*, matching the on-target efficiency of the *Vch*CAST vector alone (Fig. 3C). Furthermore, both vectors shared an equal proportion of reads that inserted at the expected site ~49 bp downstream of the protospacer adjacent motif (PAM) in *E. coli* (Fig. 3D). Analysis of transconjugants for cointegrates, where the duplicated transposon and the entire plasmid backbone are integrated, were performed by colony polymerase chain reaction (cPCR) (fig. S6, A to C) and whole-genome sequencing (fig. S6, D and E). We consistently observed equivalent cointegration rates between λ-Red *Vch*CAST and *Vch*CAST: around 12% across both treatments and analysis methods (fig. S6, C to E). The vast majority of the screened simple insert clones were observed in the transposon right-left (T-RL) orientation for both λ-Red *Vch*CAST and *Vch*CAST (figs. S6C and S6E). These data support λ-Red’s positive impact on *Vch*CAST editing efficiency without affecting insertion outcomes.

### λ-Red–assisted *Vch*CAST editing in additional Gram-negative bacteria

Motivated by the successful implementation of λ-Red to increase *Vch*CAST-directed editing efficiency in *E. coli*, we evaluated the impact of λ-Red on *Vch*CAST editing in Gram-negative bacteria with lower baseline editing efficiencies. We selected *P. putida* KT2440, a relevant strain for industrial biotechnology and bioremediation (*23*), and *K. michiganensis* M5a1*,* a plant-associated nitrogen-fixing strain and close relative to an important human pathogen (*24*, *25*). *Vch*CAST and λ-Red *Vch*CAST vectors delivering a kanamycin resistance cargo were queried for their effect on insertion efficiency (fig. S7, A to D).

**Fig. 4.**
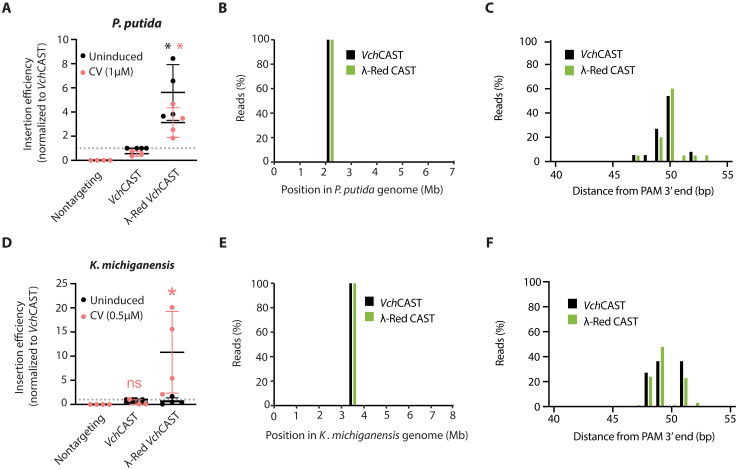
λ-Red improves *Vch*CAST editing efficiency in *P. putida* and *K. michiganensis*. (**A**) Normalized editing efficiency of *Vch*CAST compared to λ-Red *Vch*CAST in *P. putida*. CV induction was performed at 1 μM. Asterisks denote the degree of significance determined by a one-sample *t* test (one-tailed *P* value: *P ≤ 0.05; *n* = 4 biological replicates) compared to the normalized *Vch*CAST control = 1. (**B**) On-target insertion frequency across the *P. putida* genome via whole-genome sequencing. (**C**) Distribution of cargo insertion loci downstream (base pairs) of the PAM in *P. putida*. (**D**) Normalized editing efficiency of *Vch*CAST compared to λ-Red *Vch*CAST in *K. michiganensis* with CV inductions performed at 0.5 μM. (**E**) On-target insertion frequency in *K. michiganensis*. (**F**) Distribution of cargo insertion loci in *K. michiganensis.*

In both species, the presence of λ-Red on the *Vch*CAST vector yielded an increased insertion efficiency with comparable accuracy to *Vch*CAST alone (Fig. 4). We tested a range of CV induction concentrations in *P. putida* and *K. michiganensis* and selected 1 and 0.5 μM, respectively (fig. S7, E and F). There was no statistical difference between the induced and uninduced *Vch*CAST controls in both species (Fig. 4, A and D). In *P. putida*, we observed a significant increase in editing efficiency with and without the CV inducer (3.1 ± 1.2–fold and 5.6 ± 2.3–fold, respectively) compared to the uninduced *Vch*CAST control (1) (one-tailed, one-sample *t* test). Whole-genome sequencing confirmed that the edits in *P. putida* with both the *Vch*CAST and λ-Red *Vch*CAST were on-target (Fig. 4B) and followed similar insertion distribution patterns as expected (Fig. 4C). In *K. michiganensis*, we observed a significant but variable increase in editing efficiency (10.8 ± 8.4–fold) in the presence of the CV inducer (Fig. 4D). Whole-genome sequencing determined 100% on-target editing efficiency (Fig. 4, B and E) and comparable relative insertion distributions for both vectors tested (Fig. 4, C and F) for both target species. Together, these results suggest the potential of using λ-Red *Vch*CAST to enhance on-target *Vch*CAST editing efficiency for precise genome engineering in diverse Gram-negative bacteria.

### Distribution of *Vch*CAST inhibitors and activators across diverse bacteria

**Fig. 5.**
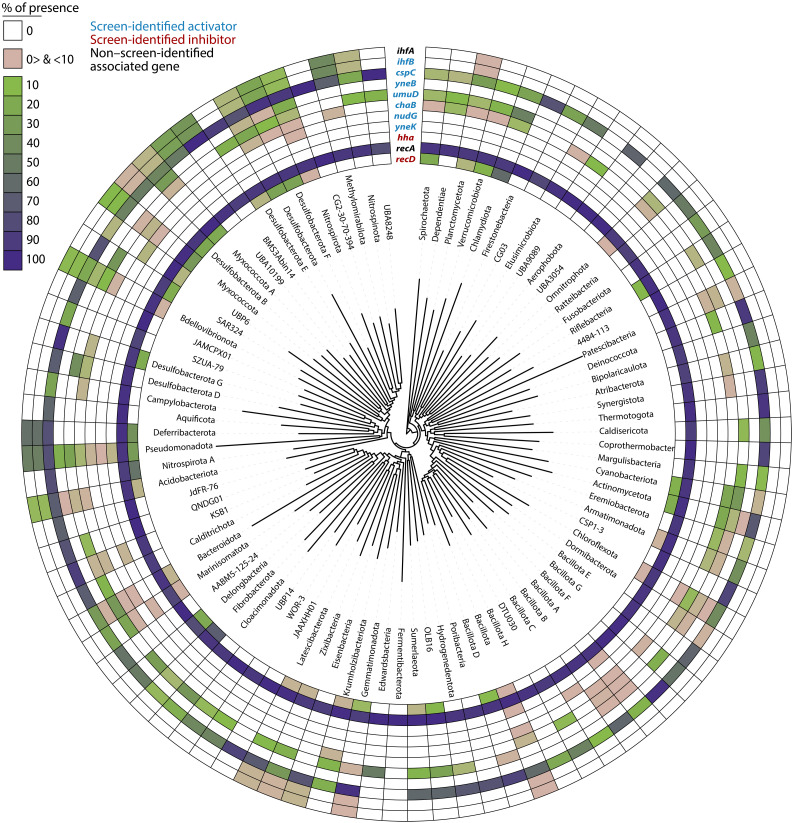
Phylogenetic distribution of *Vch*CAST activator and inhibitor genes. The phylogenetic distribution of 11 *E. coli* regulatory genes was mapped across 80,789 representative bacterial genomes from 92 phyla in Genome Taxonomy Database, release 214.0 (GTDB version 214.0) (*72*, *73*), with at least 10 members. Homologs were identified using AnnoTree (*60*) and confirmed with the eggNOG database (*70*).

To understand the broader relevance of our findings, we investigated the phylogenetic distribution of *Vch*CAST activator and inhibitor genes across the bacterial tree of life. We analyzed the presence of our seven screen-identified *Vch*CAST activators, two inhibitors, and *ihfA* (*12*) and *recA* (shown to be a strong activator in this study) across all bacterial phyla with more than 10 members in the AnnoTree database. Our analysis revealed diverse conservation patterns among these genes (Fig. 5). *recA* has a near-universal presence across the microbial tree of life and was observed in more than 90% of species in most phyla (Fig. 5). *cspC* is highly conserved across phyla (Fig. 5) but not among species with native type I-F CASTs (fig. S8). In contrast, other genes identified from the screen, such as *yneK*, *hha*, *ynbE*, and *chaB*, are absent across most phyla beyond the *E. coli*–containing Pseudomonadota and occur only sporadically within the few phyla where they are found (Fig. 5). Type I-F CAST systems are almost exclusively found in Pseudomonadota (*26*, *27*), representing just a single branch on this phylum-level tree. Notably, key regulatory genes such as *ihfA*, *ihfB*, *recA*, and *recD* occur in more than 90% of these type I-F CAST–containing genomes (fig. S8). Our analysis aims to serve as a guide for identifying where these regulators might enhance editing efficiency, particularly in taxonomic branches that naturally lack type I-F systems.

## DISCUSSION

In this study, we conducted a genome-wide mutant screen to identify genes in *E. coli* that influence the efficiency of *Vch*CAST, a promising tool for precise DNA insertion of large cargos in bacteria and eukaryotes. We screened for and then validated nine candidate genes, with eight being previously unknown, that either positively or negatively affect *Vch*CAST insertion activity when disrupted (Figs. 1B and 2A). Notably, our results highlighted a role for the RecBCD complex in CAST integration, with the disruption of *recD* increasing editing efficiency, while a *recA* deletion strongly decreased it (Fig. 2B). Building on these insights, we leveraged the bacteriophage λ-Red genes (*exo*, *beta*, and *gam*) as an alternative recombination system to enhance CAST insertion efficiency (Fig. 3A). By optimizing the expression of these genes, we achieved improved editing efficiency not only in *E. coli* by 55.2-fold (Fig. 3B) but also in *P. putida* by 5.6-fold (Fig. 4A), an industrial model strain (*23*), and *K. michiganensis* by 10.8-fold (Fig. 4D), a plant-associated nitrogen-fixing strain and close pathogen relative (*25*). This work provides a comprehensive survey of host factors influencing *Vch*CAST integration and presents an approach to enhance its efficiency across various bacterial species.

Beyond the homologous recombination–associated RecD, we found eight other activators and inhibitors of the CAST complex that may further contribute to understanding *Vch*CAST transposition (Fig. 2A). The putative activator UmuD, in complex with UmuC, performs translesion synthesis following activation by DNA damage (*28*). In the context of *Vch*CAST integration, UmuD may become activated when a replication fork collides with an unrepaired single-stranded DNA break at the integration site, allowing the replisome to continue past the unrepaired lesion (*28*). The putative activator cold shock protein C (CspC), known to bind to single-stranded nucleotides (*29**,* *30*), may stabilize the RNA guide or the single-stranded gap of the poststrand transfer intermediate. However, given that we performed all of our conjugation and editing experiments at 30°C rather than 37°C following the protocols for improved *Vch*CAST editing efficiency (*5*), it is also possible that the observation of *cspC* is an artifact as *cspC* expression and activity may be important for the fitness and viability of recipient cells at the lowered temperature. ClpX, a sequence-specific AAA+ adenosine triphosphatase with protein unfolding capabilities that facilitated more efficient editing in human cells (*10*), was not identified in our screen. It is possible that in *E. coli*, one or more partially redundant proteolytic systems such as ClpAP, ClpYQ, and the Lon protease can perform ClpX’s activity in CAST integration. Although the human proteasome provides analogous protein degradation functions, the absence of close homologs to these bacterial proteases may explain why ClpX’s addition specifically enhances *Vch*CAST function in human cells. The previously unidentified activators and inhibitors found in this study provide valuable targets for further experiments to understand and control the function of *Vch*CAST.

Among the strong activators and inhibitors identified and validated within this study, we were particularly interested in the role of the RecBCD complex. RecD was identified as the strongest inhibitor of *Vch*CAST integration via the screen and Keio mutant validation (Figs. 1B and 2A). Notably, this result is consistent with observations made for Tn*7* transposons, the native system from which all type I-F CASTs originally derived, where activity is increased in RecD-deficient *E. coli* (*31*). RecD functions as an inhibitor to homologous recombination by blocking RecA loading, and Δ*recD* mutants have been found to be hyperrecombination-proficient (*16*). Therefore, it was expected that RecA, a single-stranded binding protein essential for Rec-mediated double-stranded break repair, showed an activating phenotype when single mutants were tested for *Vch*CAST integration efficiency (Fig. 2B). On the basis of this low editing efficiency in the absence of *recA*, we would expect the decreased efficacy of *Vch*CAST in the ∆*recA* genotypes of many commercial *E. coli* strains. The weaker phenotypes of RecBC may be explained by some amount of functional redundancy in RecBCD-mediated repair compared to other repair pathways, such as the RecF homologous recombination pathway (*32*). In the Mu transposon, the RecBCD complex facilitates the repair of a double-stranded break that occurs during the resolution of the insertion (*33**,* *34*). Similarly, we hypothesize that the highly stable *Vch*CAST posttransposition complex could stall replication forks and introduce a double-stranded break at the target site, similar to Mu’s mechanism (*10*). RecBCD and RecA would then repair the double-stranded break. This putative function is supported by our finding that CIP, the DNA-damaging, SOS response, and homologous recombination–inducing agent, and λ-Red, the highly efficient homologous recombination system, improve the efficiency of *Vch*CAST. Such a homologous recombination–dependent mechanism for *Vch*CAST would contrast with the commonly stated assumption that transposition occurs independently of this DNA repair pathway (*5*, *6*, *9*, *10*, *35*). Further experiments are necessary to validate this potential role of homologous recombination and RecBCD in *Vch*CAST function.

To examine the role of RecBCD and other effector proteins in *Vch*CAST function, we delivered *Vch*CAST via a suicide vector. This approach leads to lower insertion efficiencies but provides an opportunity to examine the effects of activating and inhibiting proteins while ensuring that all selected transconjugants are bona fide edited cells. Furthermore, a suicide vector is advantageous for eventual microbiome editing applications, given that it is eliminated after cargo delivery, thus presenting fewer biocontainment concerns. The efficiencies we report (around 1 × 10^−3^ for *Vch*CAST and 5 × 10^−2^ for λ-Red *Vch*CAST; table S4) make up a small portion of the total cells but are consistent with those seen with homologous recombination (between 1 × 10^−4^ and 1 × 10^−3^ depending on the genetic background) (*36*) and transposon insertion with the transient presence of the editor (*8**,* *37*). The highest efficiencies reported in the literature are achieved in studies that deliver the *Vch*CAST on a replicating plasmid, which is then selected for, where insertions can be introduced to nearly 100% of cells (*5*, *6*, *12*, *38*). This is likely due to the presence of multiple copies of the *Vch*CAST vector persisting in cells for an extended time. Given that λ-Red has often been used to increase homologous recombination efficiency on a replicating vector (*39*), we expect that our λ-Red *Vch*CAST system would also work in that context.

Our findings on improving *Vch*CAST efficiency in *E. coli* prompted us to investigate the broader applicability of this approach in other bacterial species. When the same λ-Red *Vch*CAST system was tested in Pseudomonadota outside of *E. coli,* we found that it enhanced editing efficiencies but to a lesser extent and with more variation (Fig. 4, A and D). In *P. putida*, we observed increased editing efficiency with uninduced λ-Red *Vch*CAST (Fig. 4A). These results suggest that the leaky expression of λ-Red is sufficient to significantly increase editing efficiency, which could be due to an incompatible binding site for the repressor, EilR. In addition, we chose the CV concentration for *P. putida* on the basis of a previous characterization of pJEx in this strain (*40*) and *P. putida*’s ability to grow on CV as a sole carbon source (*41*). Incorporating analogous recombination machinery, such as the *rac* prophage–derived recombination system recET, could be a viable approach to further increase editing efficiency in genomes with higher GC content, such as *P. putida* (*42*). In *K. michiganensis*, we observed tight regulation of λ-Red via pJEx but high day-to-day variability in the effect of λ-Red *Vch*CAST (Fig. 4D), suggesting the potential for compensatory mutagenesis of λ-Red when highly expressed because of Gam toxicity (*43*). These data suggest that insights from this screen will be most efficiently applied in a host-dependent manner. For example, commandeering the recombination machinery from a phage infecting the bacteria of interest and integrating it with the *Vch*CAST vector would likely allow for more effective editing.

Beyond Pseudomonadota, where type I-F CASTs are largely confined, many of the activators identified in this study have a limited presence (Fig. 5). Notably, IHF, which is essential for efficient *Vch*CAST transposition, is absent from the majority of the bacterial tree of life. Given IHF’s importance in *E. coli*, it may be integral for targeting strains without endogenous IHF. For instance, members of Bacillota and Actinomycetota, where *Vch*CAST has shown to have low efficiency (*11*), lack close sequence homologs of IhfA and IhfB. While Actinomycetota does contain sequence-divergent but functionally homologous IHF-like proteins (*44*, *45*), their ability to support transposition is unknown. The addition of *ihfA* and *ihfB* to *Vch*CAST editing vectors in these phyla, as well as other phyla where their homologs are absent, may increase editing efficiency. These considerations highlight the relevance of tailoring optimization strategies to the specific genetic characteristics of the target organism when applying *Vch*CAST systems more broadly.

Beyond type I-F systems like *Vch*CAST, type V CAST systems show promise because of their smaller size. However, they have demonstrated significantly lower on-target specificity (*6*–*8*). In their native cyanobacterial hosts, crRNA (CRISPR RNA) matches with surrounding target sequences suggest higher accuracy (*46*). This indicates potential host-associated genes in these organisms that could enhance efficiency when the systems are applied to distant taxa (*13*). Our systematic screening approach could identify these host factors affecting type V CAST targeting precision, potentially mitigating their current limitations.

We are excited about the potential of this work to bring the powerful CAST editing toolset to more biological systems. *Vch*CAST has mostly been applied in Gammaproteobacteria (*5*, *8*, *47**–**51*). The understanding of host factors that enable and inhibit these systems is an important barrier to their use across the phylogenetic tree of bacteria. In the complex microbiomes that are most relevant for human health and the environment, editing efficiency is a major bottleneck for delivering functional cargo insertions via *Vch*CAST (*8*). This work provides strategies for more efficient delivery so that the function of these communities can be better probed and controlled at a genetic level. Overall, our findings provide insights into the factors influencing CAST efficiency and offer strategies for improving its performance across diverse organisms, paving the way for broader applications in genome editing.

## MATERIALS AND METHODS

### RB-TnSeq screen for *E. coli* regulators of CAST integration

To systematically identify *E. coli* genes that contribute to *Vch*CAST-mediated integration, we conducted a genome-wide loss-of-function mutant screen and selected for *Vch*CAST edits after conjugation in the KEIO_ML9 *E. coli* RB-TnSeq library (*14*, *52*). This approach allowed us to generate and select for *Vch*CAST edits across a diverse array of insertion mutants. The library comprises 152,018 uniquely barcoded single-gene transposon insertion mutants, covering 3744 nonessential protein-coding genes of a total of 4146 in the *E. coli* genome (*14*). To account for biases introduced by the antibiotic resistance marker used, two different antibiotic selection cargos (P_Pmtl_-*gmR* and P_Pmtl_-*catP*) were used in parallel for the RB-TnSeq and *Vch*CAST conjugation efficiency assays. A *mariner* transposase system containing matched backbones, copy control, and cargo expression cassettes were used as a control for conjugation and nonspecific transposition.

The KEIO_ML9 RB-TnSeq library was inoculated into LB containing kanamycin (25 μg/ml) and grown at 37°C with shaking. Donor strains harboring the *Vch*CAST and *mariner* vectors in *E. coli* WM3064 [diaminopimelic acid (DAP) auxotroph, *pir*+, and RP4+] (*53*) were grown at 37°C with shaking in LB containing diaminopimelic acid (DAP; 0.3 mM) and gentamicin (screen 1; 50 μg/ml) or chloramphenicol (screen 2; 34 μg/ml). Three 10 optical density (OD)*ml samples of the library overnight were washed and pelleted before being frozen at −80°C as three technical replicate time zero (*T* = 0) samples. After washing and resuspending in LB containing DAP, 1 OD*ml of donor was combined with 1 OD*ml of recipient in separate 1.5-ml Eppendorf tubes. Each combined donor-recipient sample was plated onto a plain LB agar petri plate topped with a MF-Millipore Membrane Filter for conjugation. After 6 hours of conjugation at 30°C, cells from two conjugation plates were scraped into 20 ml of LB as a single technical replicate (six conjugation plates for three technical replicates for each donor-recipient combination). One hundred microliters of the resuspended cells was set aside for 10-fold serial dilution and spot plating. One milliliter of the resuspended cells from each technical replicate was used to inoculate 100 ml of LB containing kanamycin (for the RB-TnSeq library background) and either gentamicin or chloramphenicol to select for CAST or *mariner* integrations. The three technical replicates of the library not introduced to any donor were inoculated into nonselective liquid cultures of LB with kanamycin. The liquid cultures were grown at 30°C for 12 hours with shaking. Cells from liquid cultures of each technical replicate were pelleted and resuspended in 40 ml of LB. A 2-ml aliquot of the resuspended cells was taken for genomic DNA extraction via the QIAGEN DNeasy PowerSoil Pro Kit. Barcodes were amplified with the BarSeq_v3 primers following the BarSeq (barcode sequencing) PCR protocol (*14*). Amplicons were pooled into a synthetic amplicon library and submitted for sequencing by Illumina NovaSeq PE150.

BarSeq sequencing reads were processed using the FEBA pipeline described previously (*14*). In this pipeline, the final gene fitness value was calculated by averaging the fitness scores of all strains with independent transposon insertions located in the central region of the gene, excluding those near the beginning or end. The pipeline-generated fitness scores (fit_logratios_good) were further analyzed using R (version 4.4.0) with the tidyverse package (version 2.0.0). Within an experimental trial, treatment replicates were averaged, and then the difference between the *Vch*CAST fitness scores and the *mariner* fitness scores was calculated. To identify genes consistently displaying strong differential effects across the *gmR* and *catP* insertion screens, we focused on those with an absolute fitness difference greater than one in both experimental trials. Positive fitness values generated by the FEBA pipeline indicate a putative inhibitor of *Vch*CAST, as mutating the gene increases the fitness of the strain. Conversely, negative fitness values indicate putative activators of *Vch*CAST function.

The RB-TnSeq experimental workflow is vulnerable to bottleneck effects in the library propagation and during growth in subsequent experiments. For screen 1 (gentamicin selection), we observed that 107 of 3744 genes (2.9%) with mutants in the library database were missing in the starting library at time zero (*T* = 0), including the known factor *ihfA*, suggesting that bottlenecking could have occurred during library propagation or outgrowth. Screen 2 (chloramphenicol selection) was missing only 1 of the 3744 (0.03%) nonessential genes represented in the starting library. To account for potential false negatives, a list was generated to summarize the genes that were missing from either screen that did not pass the quality control threshold for the FEBA pipeline as a result of insufficient abundance at time zero (*T* = 0) (table S2), such as *ihfA*, that could still be important for *Vch*CAST editing.

### Validating activators and inhibitors with Keio *E. coli* mutants

We next validated the involvement of putative host factors identified in the RB-TnSeq screen by performing *Vch*CAST editing in *E. coli* Keio collection deletion mutants corresponding to the genes with the largest fitness values (absolute gene fitness >1) and having molecular functions of interest as detailed on the Gene Ontology database (*54*). Factors that do not have molecular functions of interest, particularly membrane-associated factors, or those that are absent from the Keio collection were not validated or pursued in further experiments. To confirm the Keio strains, primers were designed to amplify the kanamycin resistance gene insertion within each target locus of the expected Keio mutants (table S5) (*55*). The Δ*yicI* mutant was selected as the negative control as it is a context-neutral genomic locus with no documented adverse fitness effects (*15*).

To assess how the presence or absence of individual *E. coli* genes affects *Vch*CAST integration into the target genome, we implemented a conjugation-based editing efficiency assay (fig. S1B) (*9*). Briefly, donor and recipient strains were grown for 16 hours, washed, resuspended in LB containing DAP, combined in a 1:1 ratio (0.1 OD*ml each), spotted on LB agar within a 24-well block, and allowed to conjugate for 6 hours at 30°C. Afterward, spots were resuspended in 1 ml of LB media. Ten-fold serial dilutions were performed with resuspended cells, spotted onto LB agar and LB agar with antibiotics, and incubated overnight at 30°C. Serial dilution spot plates were left to grow until individual colonies formed. Last, the plates were imaged, and cell colonies were counted to compute editing efficiency (*8*, *9*), with transconjugants reflecting successful conjugation and insertion of selective cargo. Fold change in editing efficiency was computed by normalizing the editing efficiency of candidate regulator hits of interest to that of the Δ*yicI* control. Because of day-to-day variation in editing efficiencies, normalizations were performed by pairing the treatment to the control of that day. The raw editing efficiencies of the Keio validation experiment, along with those from all experiments in the manuscript where efficiencies were normalized to a control, are reported in table S4. Three biological replicates were used for each experimental condition. A one-sample *t* test was used to determine statistical significance compared to the Δ*yicI* normalized control (hypothetical value = 1), thus asking whether the treatment condition (normalized) is significantly different than the normalized control. While multiple comparisons to the control were made, the statistical approach chosen works to compare normalized data with unmatched variance. A one-tailed *P* value is reported for each treatment on the basis of hypotheses (e.g., activator and inhibitor) generated from the genome-wide mutant screen.

### CIP-stimulated homologous recombination correlates with increased *Vch*CAST activity

We further explored the involvement of homologous recombination in *Vch*CAST integration by assessing the editing efficiency of *Vch*CAST in BW25113 *E. coli* recipients incubated with CIP, a fluoroquinolone antibiotic reported to increase homologous recombination in *E. coli*. We tested the MIC of CIP in liquid culture according to National Committee for Clinical Laboratory Standards recommendations (*56*) and observed a MIC of 160 ng/ml and one-half MIC of 80 ng/ml, corresponding to previously described concentrations (*17*).

To assess how the CIP-induced increase in homologous recombination influences *Vch*CAST integration, we performed the conjugation-based editing efficiency assay with some minor changes (fig. S1B). After 16 hours of growth, MIC CIP (160 ng/mL) and one-half MIC CIP (80 ng/ml) were added to overnight cultures of select BW25113 recipients and then allowed to incubate for two additional hours (*17*). Fold change in editing efficiency was computed by normalizing the editing efficiency of CIP-exposed recipients to that of the no-CIP control. Three biological replicates were used for each experimental condition. A one-sample *t* test was used to determine statistical significance compared to the normalized control (1). A one-tailed *P* value is reported for the experimental treatments on the basis of the hypothesis that increasing homologous recombination would increase editing efficiency.

### Construction and testing λ-Red *Vch*CAST in *E. coli*

We constructed versions of the *Vch*CAST vector that included different permutations of the λ-Red recombineering system (*exo*, *beta*, and *gam*) onto the backbone. The DNA of λ-Red and Jungle Express (pJEx) were purchased as gblock gene fragments (IDT) and added to *Vch*CAST vectors using Gibson and Golden Gate assemblies. The sequence for λ-Red was sourced from the BioDesignER *E. coli* strain to maximize recombination efficiency while reducing toxicity (*15*). We cloned λ-Red onto a *Vch*CAST backbone just after the origin of transfer, ensuring quick transcription in the recipient cell during conjugation. Our experimental design deliberately uses nonreplicative vectors (R6K origin of replication) to maintain a system with a sufficient dynamic range to detect both activating and inhibiting effects. The tightly regulated and strong, inducible pJEx was used to minimize leaky λ-Red expression and toxicity of the vector (*43*). pJEx is inducible with CV, and transcriptional control is robust in Pseudomonadota (*40*). Plasmids, strains, synthesized DNA, and oligonucleotides (IDT, Coralville, US) used in the study are listed in the Supplementary Materials (tables S5 to S7). *Vch*CAST vectors were assembled through multipart Golden Gate cloning. High-fidelity PCRs were performed with Q5 Hot Start High-Fidelity DNA polymerase (NEB). Golden Gate assembly enzymes (e.g., BsmBI-V2, BbsI, BsaI-HFV2, and T4 ligase) were ordered from NEB and used with the reported buffers following previously reported protocols (*8*). *Vch*CAST assemblies were electroporated into electrocompetent *E. coli* EC100D*pir*+ cells (LGC Biosearch). Clones were screened by cPCR with 2× GoTaq Green Mastermix (Promega), and plasmids were isolated with a QIAprep Spin Miniprep Kit (Qiagen). Guide assemblies were electroporated into *E. coli* WM3064 *pir*+ and grown on the appropriate antibiotics and DAP. All vectors were confirmed with whole-plasmid sequencing (Plasmidsaurus Labs).

CV induction was tested in *E. coli* to determine the concentration that produces the highest insertion efficiency (fig. S5). Conjugations were performed on agar plates containing the CV at the optimal induction concentration (0.01 μM) for 6 hours before resuspension and selection. To account for variability in editing efficiency between biological replicates, induced λ-Red VchCAST treatments were paired and normalized to induced VchCAST control. Each experiment was repeated with three biological replicates. A one-sample *t* test was used to determine statistical significance compared to the normalized control (1). A one-tailed *P* value is reported for the experimental treatments on the basis of the hypothesis that introducing an alternative recombination machinery would increase *Vch*CAST editing efficiency compared to the control.

### Testing λ-Red *Vch*CAST in *P. putida* and *K. michiganensis*

We first performed a quantitative assay in candidate strains to determine the frequency of mutants resistant to the antibiotics streptomycin/spectinomycin/carbenicillin (100 and 400 μg/ml), chloramphenicol (34 and 68 μg/ml), kanamycin (25, 50, 100, and 200 μg/ml), and gentamicin (10, 20, and 40 μg/ml). Overnight cultures of each species were grown in LB at 30°C, 10× serially diluted, and spotted onto LB agar without antibiotics (control) and onto each of the antibiotic concentrations. Colonies were counted after 16 to 40 hours of growth, and the antibiotic concentration exhibiting minimal or no detectable growth was chosen as a selection marker for genome editing experiments (fig. S7, A and B). On the basis of these results, we constructed a *Vch*CAST vector containing the P_pmtl_ promoter driving the kanamycin resistance gene for selection in *P. putida* and *K. michiganensis*.

We identified safe sites and designed guides in *P. putida* (GCF_000007565.2) following previously reported methods (*8*, *9*) and used a previously tested safe site guide in *K. michiganensis* (*8*). Briefly, intergenic regions between converging genes with a distance of 300 to 600 nucleotides were selected (fig. S7D). Candidate safe sites were excluded under any of the following circumstances: The region was located within or adjacent to predicted mobile genetic elements, the region was flanked by essential genes (inspected using BioCyc), or the region contained noncoding RNA features (inspected using Rfam). Within the selected safe site regions, PAMs with the sequence 5′-CN-3′ were identified. For each PAM, 32 nucleotides were added to generate a list of potential guides, which were chosen on the basis of having a GC content of 40 to 60% and ensuring that the insertion loci, ~49 bp downstream of the guide, remained within the intergenic region and that it would not accidentally disrupt a terminator sequence. Three guides per safe site were selected, and their off-target potential was assessed using a local BLASTn (Nucleotide Basic Local Alignment Search Tool) search (-dust no -word_size 4). Guides with off-target hits exhibiting the highest *e*-values and with minimal complementarity to off-targets in the seed region (first ~10 nucleotides) were prioritized. Two guides targeting different safe sites were cloned and tested (fig. S7C).

Conjugation experiments in *P. putida* and *K. michiganensis* were performed following the protocol described for *E. coli* with some modifications. Recipient and donor cells were grown for 16 hours at 30° and 37°C, respectively. Conjugations were done for 20 hours at 30°C with CV induction concentrations of 0.5 and 1 μM used for *K. michiganensis* and *P. putida*, respectively (fig. S7, E and F). Transconjugants were selected on LB plates containing kanamycin (50 μg/ml). Spot plates of 10 μl for each serial dilution were plated to increase sensitivity and reduce technical error. Three biological replicates were performed across different days. A one-sample *t* test was used to determine statistical significance compared to the normalized uninduced *Vch*CAST control (1). A one-tailed *P* value is reported for the experimental treatments on the basis of the hypothesis that increasing homologous recombination would increase editing efficiency.

### Insertion analysis in *E. coli*, *P. putida*, and *K. michiganensis*

Insertion analysis was preliminarily screened by cPCR on transconjugants following selection in all strains. Insertion orientation primers were designed to identify clones with right-left (T-RL) and left-right (T-LR) simple insertion, cointegration, and no integration outcomes (fig. S6, A and B) (*9*). Cointegration oligos were designed to amplify the junction between the *Vch*CAST vector backbone and the resistance marker in the cargo. Simple insert oligos were designed to amplify from the genomic DNA on either side of the insertion site to capture the full *Vch*CAST cargo insert. Another set of oligos was designed to distinguish between T-RL and T-LR simple insert products. Additional screening for insertion cointegration versus simple insertion was performed for *E. coli* by patch plating of transconjugants on LB agar plates containing carbenicillin (100 μg/ml), the *Vch*CAST vector backbone resistance marker. Carbenicillin resistance in *P. putida* and *K. michiganensis* did not permit cointegrate screening by double selection and analysis by cPCR.

Insertion products from all recipient strains (*E. coli*, *P. putida*, and *K. michiganensis*) and CIP treatments (MIC and one-half MIC) in this study were further assayed for off-targets by whole-genome sequencing. To address colony heterogeneity, which has been observed previously (*9*), about a thousand colonies of transconjugant cells transformed with *Vch*CAST and λ-Red *Vch*CAST were scraped from selection plates and resuspended. Three biological replicates were sequenced separately for *E. coli*. Colonies were scraped from selection plates and resuspended in a volume of LB media equivalent to OD_600_ = 3 to 4. An aliquot of this resuspension was used for high-molecular-weight genomic DNA extraction using the MasterPure Complete DNA and RNA Purification Kit (Biosearch Technologies). Genomic DNA samples were submitted for Oxford Nanopore long-read sequencing to Plasmidsaurus Labs.

The bioinformatics analysis was performed using custom scripts. The demultiplexed raw reads were filtered using the Nanofilt Python package (version 2.8.0) to select reads with a Phred quality score of 20 (>Q20) and a minimum length of 150 bp (*57*). We focused our analysis on reads exceeding 10,000 bp to guarantee the inclusion of complete cargo and insertion site information. Shorter reads, which were abundant in our dataset, were often fragmented and thus unsuitable for this purpose. To identify the inserted cargo, the reads were mapped to the complete sequence of the transposon using Blastn (*58*), and reads mapping to less than 80% of the right end of cargo were filtered out.

For downstream analysis, a pipeline developed by Vo *et al.* (*5*) was used with some modifications. The reads flanking the mapped region were extracted and aligned with the complete reference and plasmid genomes. The reads were classified as genomic, plasmid, or both on the basis of whether more than 100 bp was mapped to the respective genomes. The read classification was further validated by Bakta annotation software (*59*) to confirm the presence and correct annotation of genes associated with plasmids and genomic reference.

Last, the position of the transposon’s right and left ends as well as the PAM and safe site was determined by Blastn (*58*). Transposon orientation (RL or LR) was assigned on the basis of the distances between the right and left ends and the PAM/safe site (fig. S6, D and E). The genomic coordinates of the mapped region were recorded to generate the genome-wide histograms of the integration locations. The mapped region was categorized as on-target if it fell within the 100-bp window of the 3′ end of the target site.

### Bioinformatic homology search for regulators

We examined the phylogenetic distribution of 11 genes across 92 bacterial phyla, which included the 9 identified and validated by the RB-TnSeq screen and *ihfA* and *recA*. To simplify viewing, only phyla with more than 10 members in the AnnoTree database (v beta, based on GTDB R214.0) (*60*) were included in this analysis. Briefly, the genes in the AnnoTree database were annotated using PFAM version 27.0 (*61*), TIGRFAM version 15.0 (*62*), and Kyoto Encyclopedia of Genes and Genomes (KEGG) orthology identifiers assigned to the UniRef100 database (*63*). The KEGG or PFAM annotation assigned to individual genes was used to extract homologs from 80,789 representative genomes from GTDB R214.0 (*64*). Genes such as *ihfB*, *recD*, and *umuD* are found in close association with their respective enzymatic complex subunits, *ihfA*, *recBC*, and *umuC* (*65**–**67*). To maintain consistency, the homologs of these co-occurring genes were extracted using the same methods as for the genes of primary interest, and only genomes containing both sets of genes in the same contig were retained for further analysis. The gene *yneK* lacks a PFAM or KEGG annotation. To address this, protein sequences from the 80,789 representative genomes in the GTDB database (*64*) were downloaded and subjected to BLASTp (Protein Basic Local Alignment Search Tool) analysis against the *E. coli yneK* sequence. Proteins with greater than 50% sequence similarity and a significant FASTA hit (*E* ≤ 1 × 10^−5^) were retained. To further validate our findings, a BLASTp search was conducted against a UniProt database subset containing sequences sharing at least 90% similarity to *E. coli* yneK. Both approaches yielded comparable results. While the yneK gene is prevalent among closely related *E. coli* and *Shigella* strains, only a limited number of these strains are present in the 80,789 representative genomes in the GTDB database. The *cspC* gene is a member of the bacterial cold shock protein family (*68*), which can contain multiple paralogs with a high sequence similarity (>70%) (*69*). To accurately differentiate these paralogs, cspC proteins identified by AnnoTree were reanalyzed using eggNOG-mapper version 5.0 (*70*). This tool leverages evolutionary relationships to distinguish between proteins within the cold shock protein family excluding non-*cspC* family members. Last, the gene presence per phyla was visualized in iTOL version 6 (*71*) with the publicly available GTDB R214.0 bacterial tree inferred from the concatenation of 120 proteins (*72*, *73*).

To identify genomes having type I-F CAST, a systematic review of published literature was conducted. A dataset of 1064 genomes containing type I-F CAST, derived from three key studies (*26*, *27*, *74*), was compiled for subsequent bioinformatic analysis. Protein-coding genes were annotated using Prokka (*75*), and functional annotation was achieved through KEGG pathway mapping with kofamscan (*76*). Following the annotation method developed above, KEGG IDs were used to retrieve annotations for nine genes, eggNOG-mapper was used for *cspC*, and BLASTp was used to identify *yneK*.
