# Supplementary material for: Identification of proteins influencing CRISPR-associated transposases for enhanced genome editing
Source: Sci Adv. 2026 Jan 1;12(1):eaea1429. doi: 10.1126/sciadv.aea1429 (PMC12757027; doi:10.1126/sciadv.aea1429)
Supplement: Supplementary file 1 — Figs. S1 to S8 Legends for tables S1 to S7 [file sciadv.aea1429_sm.pdf]

Science Advances
AAAS

Supplementary Materials for

Identification of proteins influencing CRISPR-associated transposases for enhanced genome editing

Leo C. T. Song *et al.*

Corresponding author: Benjamin E. Rubin, brubin@berkeley.edu

Sci. Adv. **12**, eaea1429 (2026)
DOI: 10.1126/sciadv.aea1429

The PDF file includes:

Figs. S1 to S8
Legends for tables S1 to S7

Other Supplementary Material for this manuscript includes the following:

Tables S1 to S7

SUPPLEMENTAL FIGURES

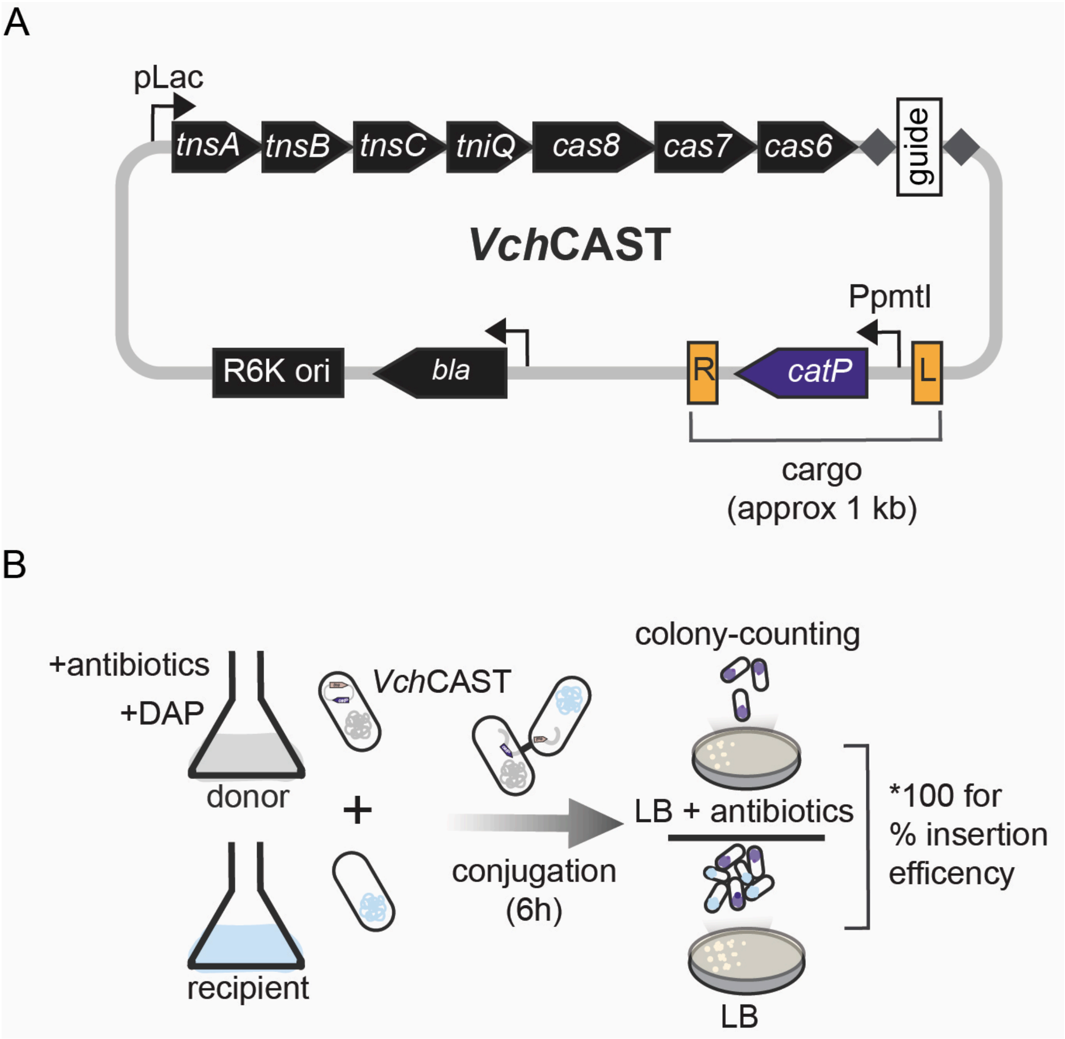

Figure S1. *Vch*CAST construct and conjugative delivery-insertion efficiency assay. (A) Plasmid schematic for the standard type I-F *Vch*CAST editing vector. (B) Conceptual schematic for conjugation-based *Vch*CAST insertion efficiency assay.

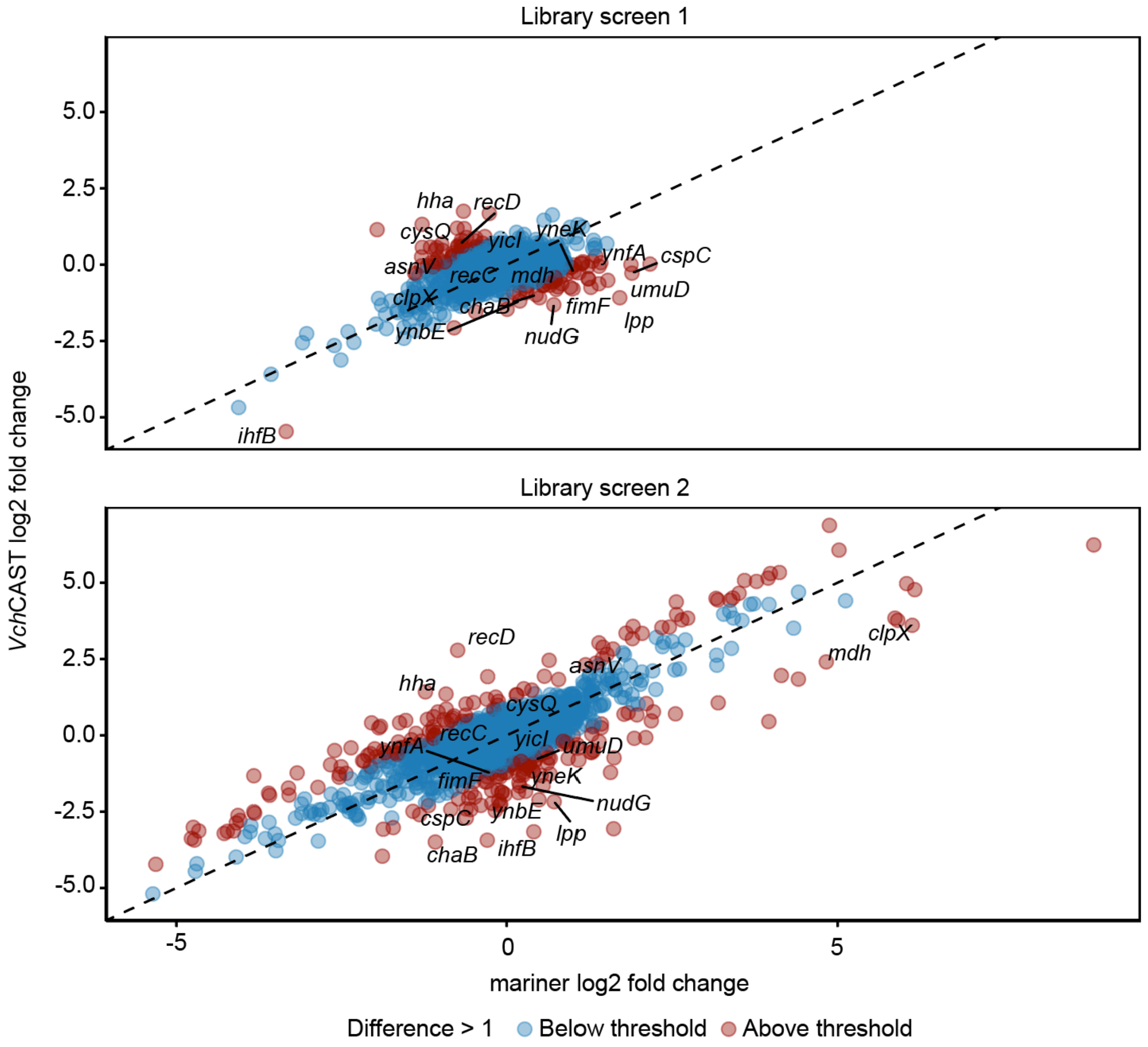

Figure S2: Comparison of relative fitness scores for single-gene insertion mutants generated using *mariner* and *Vch*CAST systems. Scatterplots display the $\log_2$ fold change in fitness from two independent RB-TnSeq library screens: screen 1 (top) and screen 2 (bottom). Each point represents the mean fitness score of technical replicates for a given gene. Points colored red indicate an absolute difference in fitness scores greater than 1 between the two systems (|*mariner* – *Vch*CAST| > 1), while blue points fall below this threshold. The dashed diagonal line represents the line of identity (y = x). Genes of interest are labeled.

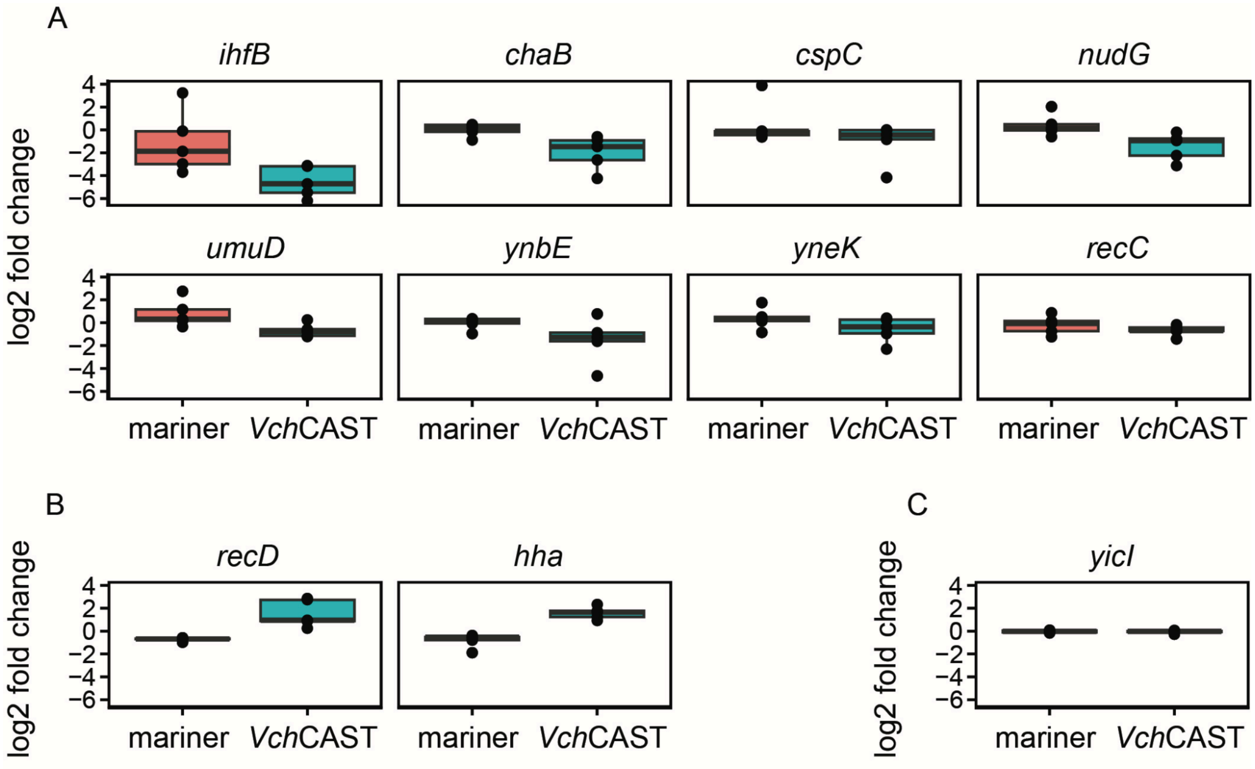

Figure S3. Comparison of fitness scores for mutants generated by *Vch*CAST and *mariner* transposon systems. Side-by-side box plots display the $\log_2$ fold change in fitness for selected genes across both *Vch*CAST and *mariner*-edited mutant libraries, aggregated from two independent RB-TnSeq screens. Each panel represents an individual gene, with points indicating replicate measurements. Genes are categorized into (A) Activators, (B) Inhibitors, and (C) Neutral/Control, based on their putative roles in the phenotype of interest.

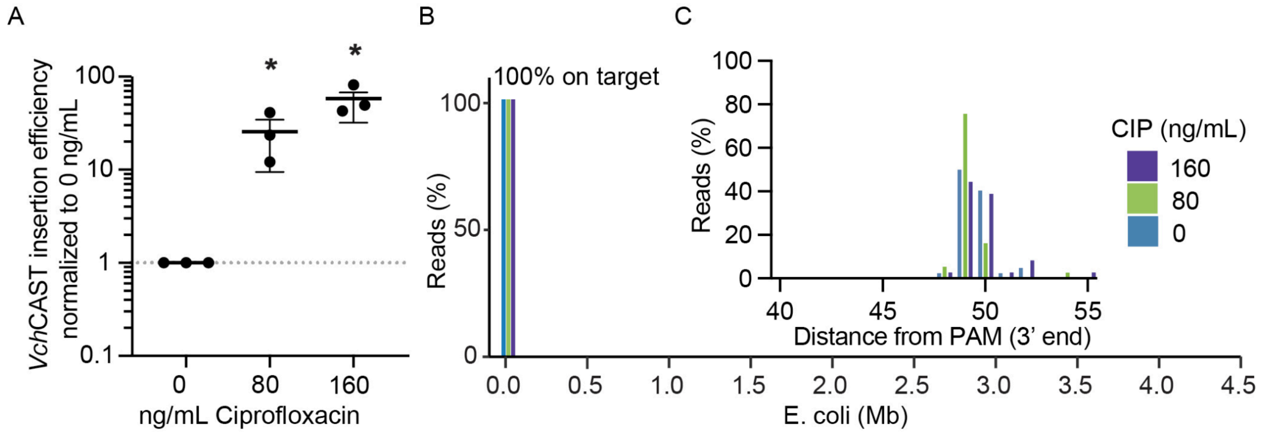

Figure S4: Ciprofloxacin (CIP) exposure increases *Vch*CAST editing efficiency in *E. coli*. (A) *Vch*CAST's editing efficiency in *E. coli* BW25113 incubated with full (160 ng/mL) and one-half (80 ng/mL) MIC CIP is normalized to that of the no CIP (0 ng/mL) treatment = 1. Asterisks denote the degree of significance determined by one-sample t-test (one-tailed p-value: * $P \leq 0.05$; $n = 3$ biological replicates). (B) On-target insertion frequency of CIP-exposed *E. coli* transconjugants via whole genome sequencing. (C) WGS read distribution (%) of insertions downstream (bp) of PAM target site.

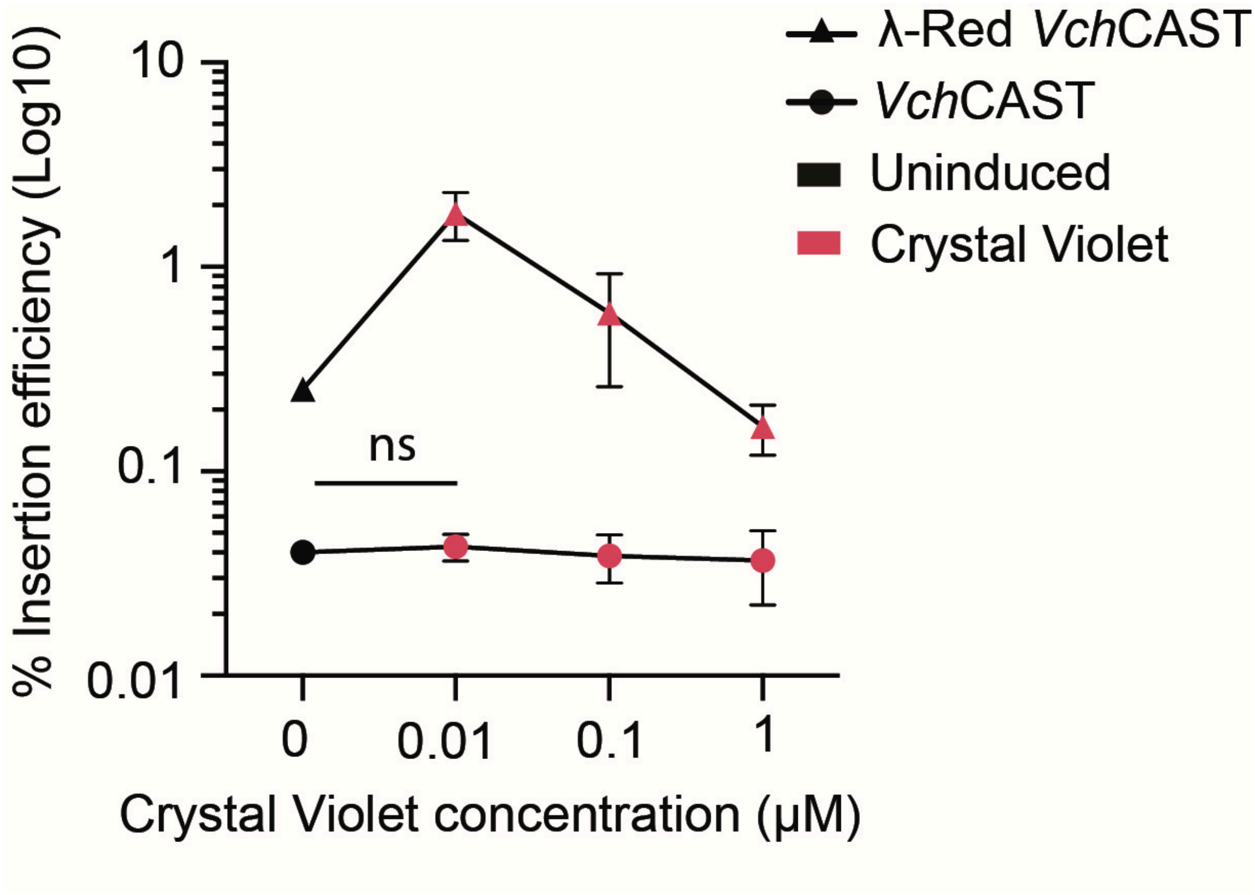

Figure S5. CV induction optimization for λ-Red expression in *E. coli*. Percentage of insertion efficiency is reported in Log10. A Kruskal-Wallis test with multiple comparisons was used to determine if any significant differences were observed across the CV concentrations (*n* = 3 biological replicates). Ns denotes not significant. Pink is used to denote treatments with crystal violet induction. Triangles represent the λ-Red *Vch*CAST treatments, while circles represent the *Vch*CAST treatment.

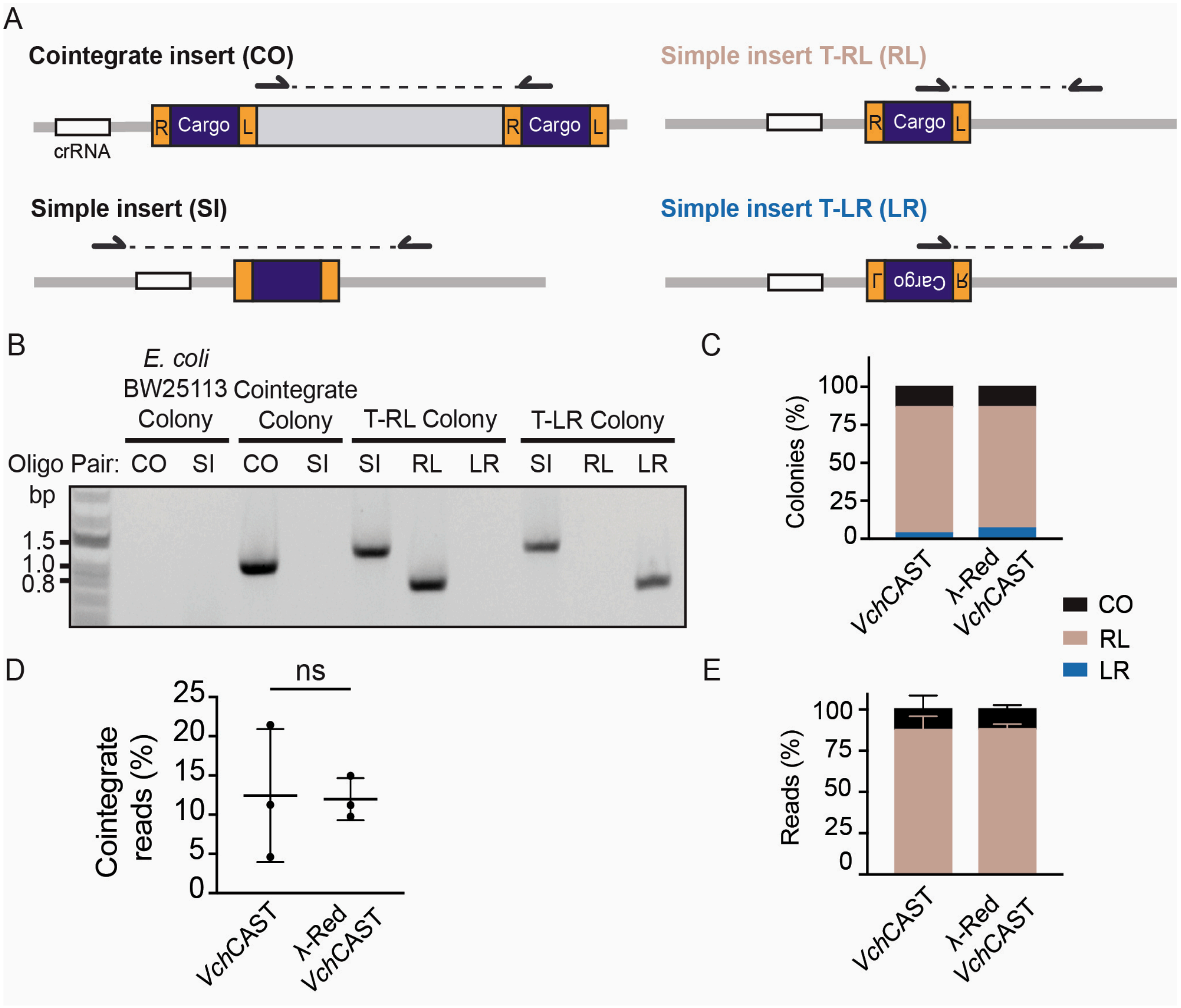

Figure S6. Comparison of insertion products for *Vch*CAST and λ-Red *Vch*CAST in BW25113 *E. coli*. (A) Schematic of primer binding and amplification for insertion product verification and orientation analysis by colony PCR (cPCR). (B) Representative gel image of cPCR analysis of *Vch*CAST insertion products in *E. coli* BW25113. Oligo pairs used for amplification are detailed in part A of this figure. CO is cointegrate, SI is simple insert, and the orientations are denoted as RL (right-left) and LR (left-right). (C) Side-by-side comparison of product type and orientation (%) of *Vch*CAST and λ-Red *Vch*CAST in *E. coli* BW25113 as characterized by cPCR. (D) Percentage of cointegrate reads determined by whole genome sequencing across three sequencing runs (*n* = 3 biological replicates). A welch's T-test determined no significant difference (ns). (E) Side-by-side comparison of product type and orientation (%) of insertion in *E. coli* BW25113 as characterized by whole genome sequencing.

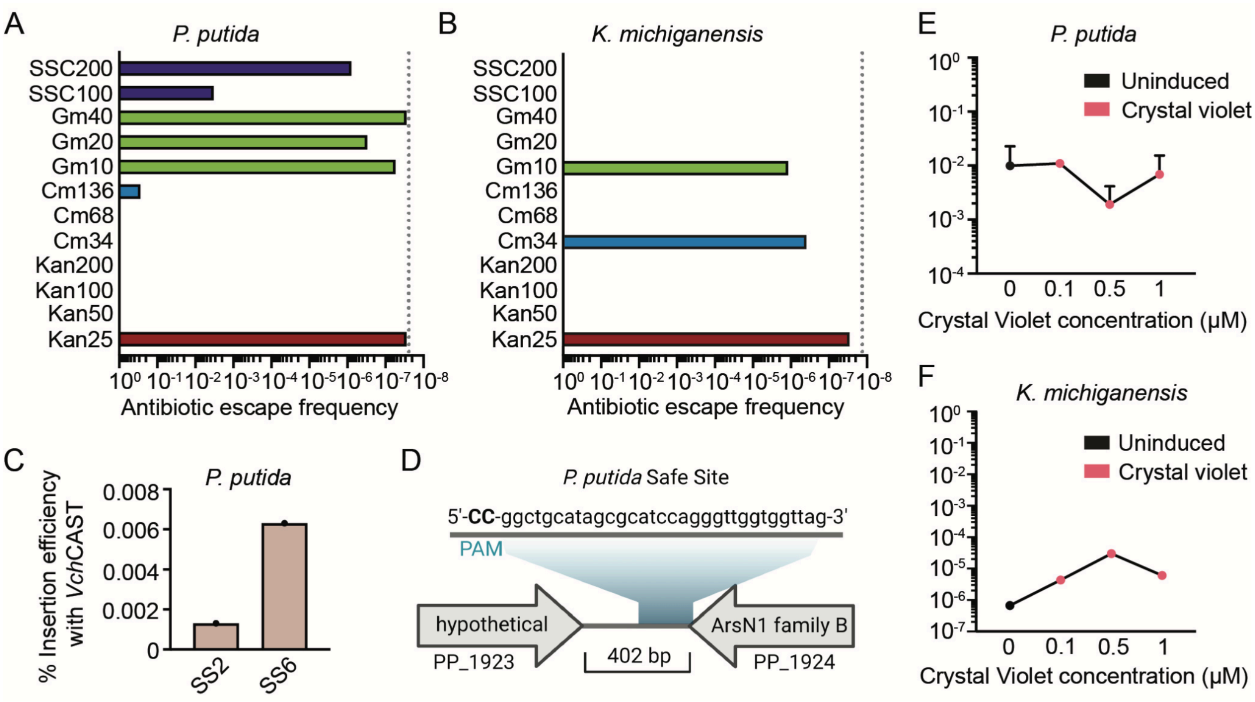

Figure S7: *P. Putida* and *K. michiganensis Vch*CAST editing. (A) Antibiotic susceptibility testing for *P. putida*. The dotted line depicts the limit of detection $2.56E^{-8}$ (B) Antibiotic susceptibility testing for *K. michiganensis*. The dotted line depicts the limit of detection $1.33E^{-8}$ (C) Safe site guide insertion efficiency (%) in *P. putida*. (D) Guide design for *P. putida* Safe site 6 (SS6). Created in BioRender. Alker, A. (2025) https://BioRender.com/m51i476. (E) CV induction optimization for λ-Red expression in *P. putida* (*n* = 3 biological replicate). (F) CV induction optimization for λ-Red expression in *K. michiganensis* (*n* = 1 biological replicate).

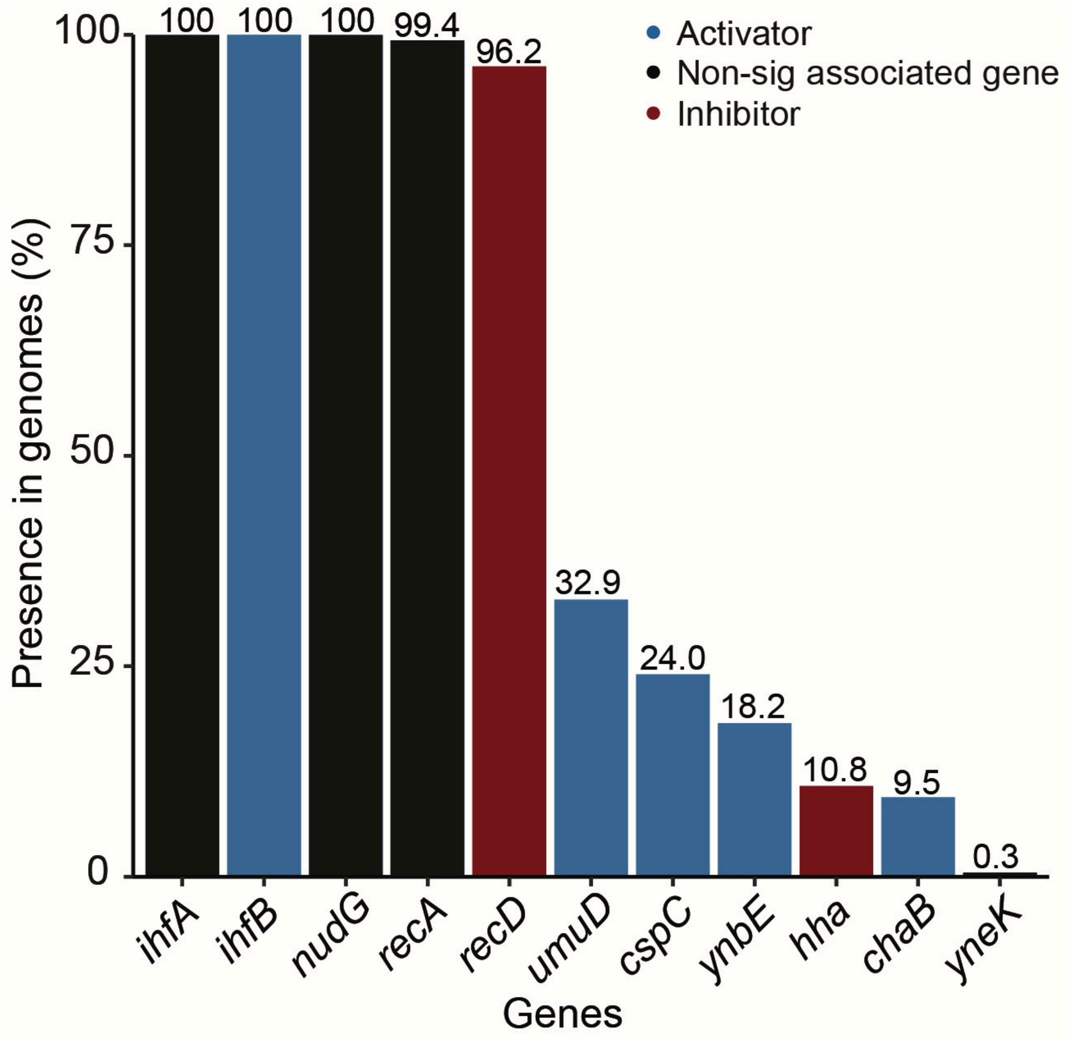

Figure S8: Percent distribution of 11 *Vch*CAST activator and inhibitor genes across type I-F CAST-containing genomes (*n* = 1069) compiled from Rybarski et al. (2021), Peters et al. (2017), and Klompe et al. (2023).

AUXILIARY SUPPLEMENTAL MATERIALS (EXCEL TABLES)

Table S1

Raw fitness scores from screens.

Table S2

List of genes absent from screens.

Table S3

Gene ontology.

Table S4

Raw editing efficiencies.

Table S5

Primers and gBlocks.

Table S6

Plasmids.

Table S7

Strains.
